# Distinct Effects of Copper Ionophores on Intracellular Copper and Cell Viability in Wild-Type and CTR1-Deficient SH-SY5Y Cells

**DOI:** 10.1021/acsomega.6c04498

**Published:** 2026-08-10

**Authors:** Anette Reinapu, Sigrid Kirss, Vello Tõugu, Peep Palumaa

**Affiliations:** Department of Chemistry and Biotechnology, 54561 Tallinn University of Technology, Akadeemia tee 15, Tallinn 12618, Estonia

## Abstract

Copper homeostasis is essential for neuronal function, and its dysregulation has been implicated in neurodegenerative disorders. In particular, reduced intracellular copper levels alongside with elevated extracellular copper concentrations have been associated with Alzheimer’s disease (AD). Therefore, strategies aimed at restoring intracellular copper levels represent a potential therapeutic approach. In this study, we investigated the effects of selected copper-binding compounds—α-lipoic acid (LA), elesclomol (ES), disulfiram (DSF), thiourea (TU), and triapine (3-AP)—on copper uptake and cytotoxicity in SH-SY5Y wild-type (WT) and CTR1-deficient (CTR1^–/–^) cells. Intracellular copper levels were quantified using inductively coupled plasma mass spectrometry (ICP-MS), and cell viability was assessed by using propidium iodide assay under various treatment conditions. Among the tested compounds, LA, ES, and DSF significantly increased intracellular copper levels in both WT and CTR1^–/–^ cells, whereas TU and 3-AP did not enhance copper accumulation beyond copper-only treatment. Notably, CTR1^–/–^ cells exhibited reduced sensitivity to copper-associated toxicity compared to WT cells. Under copper-supplemented conditions, DSF and ES displayed concentration-dependent cytotoxicity, while LA and TU induced only mild effects. In contrast, 3-AP toxicity was abolished in the presence of copper. Furthermore, cotreatment with LA and copper increased cell proliferation and promoted differentiation-associated morphological changes in CTR1^–/–^ cells. Overall, our results demonstrate that copper ionophores can enhance intracellular copper levels dependently and independently of CTR1, with distinct effects on cell viability. Among the tested compounds, LA and DSF showed a favorable profile by increasing copper levels while maintaining low cytotoxicity, highlighting their potential for modulating cellular copper homeostasis.

## Graphical abstract

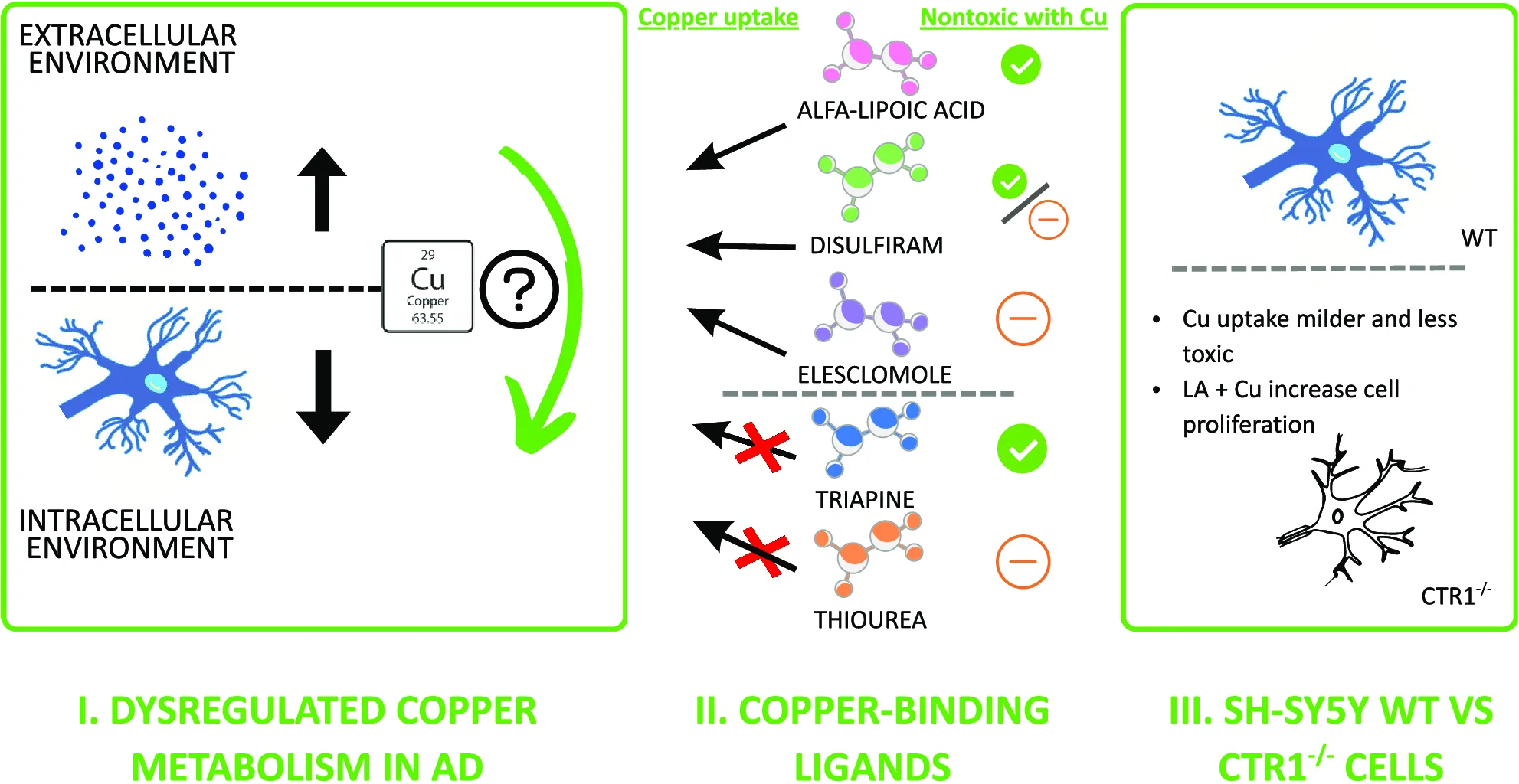

## Introduction

Copper is a vital microelement in all living organisms, and its concentration in cells must be tightly regulated, as both deficiency and excess can lead to severe physiological consequences.^1^ Copper uptake into cells is primarily mediated by copper transporter 1 (CTR1). Once inside the cells, copper is transiently bound by different specific copper chaperones, including CCS, COX17, and ATOX1, which deliver copper to target enzymes such as Cu,Zn-SOD, cytochrome c oxidase (CCO), and copper transporters ATP7A/B.^2^

Copper excess and deficiency lead to Wilson’s and Menkes disease (WD and MD, respectively).^3^ In addition, imbalances in extracellular and intracellular copper concentrations are linked to several neurodegenerative disorders, including Alzheimer’s disease (AD).^4^ AD is primarily characterized by cerebral accumulation of extracellular amyloid plaques and intracellular neurofibrillary tangles (NFT) long before clinical symptoms appear. There are many competing hypotheses about the causes of AD, including the dysregulation of copper homeostasis. Cu^2+^ can form a relatively stable complex with β-amyloid peptide (Aβ), a primary component of extracellular amyloid plaques. Aβ-Cu^2+^ complex further promotes Aβ aggregation and contributes to peptide toxicity.^5^ For AD patients, the overall copper content in brain tissue has decreased compared to healthy controls, and at the same time, copper concentration in plasma and serum is increased.^6^ Since both, the intracellular copper deficiency and extracellular copper overload may be detrimental, there is a need for normalizing copper levels by shifting extracellular copper back into cells.^7^ This can be done by ionophores, a class of compounds with the ability to bind ions noncovalently and carry them across biological membranes.^8^

α-lipoic acid (LA) is a natural compound with strong antioxidant properties that can cross the blood-brain barrier.^9,10^ Therefore, it has been proposed as a promising candidate for AD prevention.^7,9^ Previous studies have shown that LA can bind Cu^+^ ions,^11^ promote the transport of extracellular copper into cells,^12^ and protect cells from oxidative stress associated with copper overload.^13^ Compared to other copper chelators and ionophores, LA exhibits more controlled copper trafficking into cells, suggesting a favorable safety profile for normalization of copper metabolism in AD.^7^ In addition, LA has been studied in the context of AD as an antioxidant, and a combined treatment with LA and omega-3 fatty acids may help slow cognitive and functional decline in patients with mild to moderate AD.^14^ In this study, we aim to understand how LA affects cells with copper deficiency and to compare these results with other copper ionophores to establish the essential properties for efficient copper transport.

Elesclomol (ES) is a copper ionophore used primarily for its anticancer effects. The cosupplementation of ES and copper causes copper overload in cells, whereas ES alone leads to the degradation of ATP7A.^15^ ES can induce copper-mediated cytotoxicity, called “cuproptosis”, which is a copper-based form of programmed cell death.^16^ ES has been studied in the context of different types of cancer and Menkes disease.^15^ Disulfiram (DSF) is a copper ionophore, which can also cause cuproptosis at high doses.^17^ DSF is a well-tolerated drug for the treatment of alcoholism, by acting through blocking the oxidation of acetaldehyde.^18^ Thiourea (TU) forms a redox-inactive complex with copper. TU is unable to compete with histidine for Cu^2+^ binding, however, it can compete for Cu^+^ binding inside the cells.^19^ Triapine (3-AP) is a promising anticancer drug and a chelator for metal ions such as copper, iron, zinc, etc. 3-AP inhibits ribonucleotide reductase (RNR) and causes cell death; however, the addition of copper in 1:1 stoichiometric ratio can cancel the cytotoxicity of 3-AP.^20^ The decreased cytotoxicity of 3-AP when coadministrated with copper could be attributed to the high stability and low electrochemical activity of the complex Figure 1.^21^

**Figure 1:**
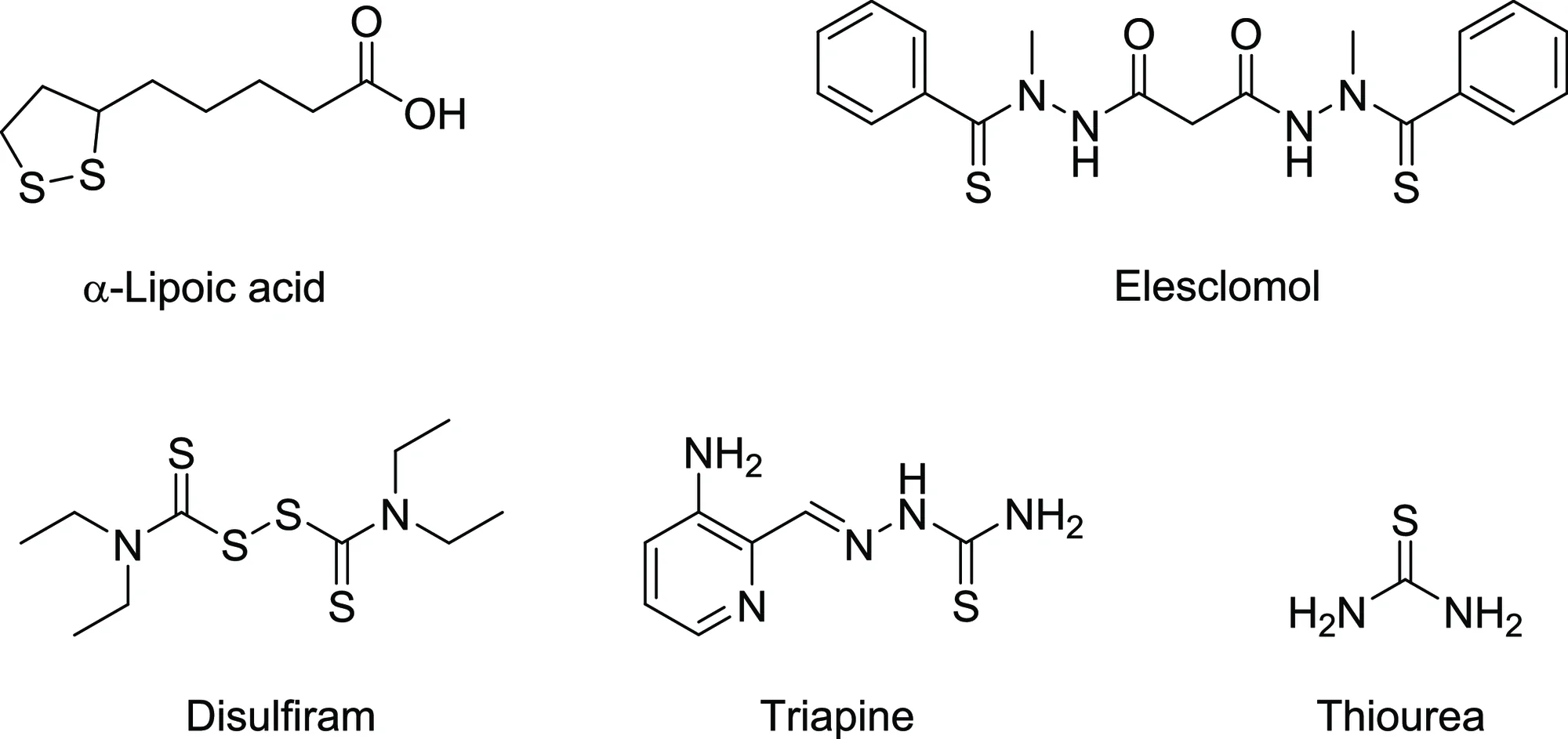
Structures of studied copper-binding compounds.

The distinct effects of the tested compounds on intracellular copper accumulation and cytotoxicity may partially arise from differences in their copper oxidation-state preferences and redox properties. Reduced form of LA has been shown to preferentially coordinate Cu^+^, thereby potentially facilitating intracellular copper trafficking while limiting redox-active copper toxicity.^11^ In contrast, ES primarily complexes Cu^2+^ extracellularly and transports copper into cells, where intracellular reduction and mitochondrial accumulation have been associated with cuproptosis induction.^16^ DSF forms lipophilic copper complexes that efficiently shuttle copper across cellular membranes. In intracellular conditions, DSF is rapidly reduced to diethyldithiocarbamate (DDC), which can bind Cu^+^ yielding stable Cu^+^-DDC complex.^22,23^ TU, although capable of interacting with Cu^+^, forms relatively redox-inactive complexes. 3-AP can chelate both Cu^2+^ and Cu^+^; however, the resulting copper complexes are relatively stable and exhibit lower redox activity.^21^

Here we describe the cytotoxicity of selected copper ionophores and chelators described above when coadministered with copper, as well as their ability to transport extracellular copper into cells. The copper trafficking ability was tested in SH-SY5Y wild-type (WT) cells and in SH-SY5Y CTR1^–/–^ (CTR1^–/–^) cells, which lack the functional copper transporter, modeling the low intramolecular copper concentration characteristic of AD. Due to compound-specific toxicity profiles, experimental concentrations were optimized individually to assess copper-dependent cellular effects under physiologically relevant, nontoxic conditions.

## Materials and Methods

### Cultivation of Cells

SH-SY5Y cells are widely used as an in vitro model in AD research due to their human origin and ability to differentiate into neuron-like cells. Following differentiation, these cells exhibit neuronal morphology and functional characteristics, making them suitable for studying mechanisms associated with neurodegeneration, including metal dyshomeostasis and copper-associated toxicity.^24,25^

Human SH-SY5Y neuroblastoma wild type (ATCC) and CTR1^–/–^ (ATCC; Faundez Lab) cell lines were cultured at 37 °C in a humidified incubator with 5% carbon dioxide. Both cell lines were grown in Dulbecco’s Modified Eagle Medium without Phenol Red (DMEM; 21063–029, Gibco) supplemented with 10% fetal bovine serum (FBS; A5256701, Gibco) and 1% penicillin and streptomycin (PEST; 15140, Gibco). The medium was changed every 2–3 days, and the cells were passaged every 5–7 days using 0.25% Trypsin-EDTA (25200, Gibco) for a maximum of 20 passages.

Depending on the experiment and the cell line, cells were seeded at densities specified in Table 1. Cell differentiation was performed using a previously established retinoic acid/BDNF-based protocol,^26^ which has been extensively validated for the induction of neuron-like morphology in SH-SY5Y cells. Briefly, cells were treated with 10 μM retinoic acid (RA; R2625, Sigma-Aldrich) in complete medium for 4 days, followed by treatment with 50 ng/mL brain-derived neurotrophic factor (BDNF; B-250, Alomone Laboratories) in serum-free medium for 2 days.^7^

**Table 1:** Cell Cultivation Plates and Initial Cell Densities used in Experiments and Cell Line

Experiment	Cell line	Cell culture plate	Initial cell density (cell/mL)
Toxicity experiments	SH-SY5Y WT	96-well plate	1,5 × 10^5^
Toxicity experiments	SH-SY5Y CTR1^–/–^	96-well plate	1,5 × 10^5^
ICP-MS	SH-SY5Y WT	6-well plate	1,0 × 10^5^
ICP-MS	SH-SY5Y CTR1^–/–^	6-well plate	1,0 × 10^5^
CTR1^–/–^ proliferation	SH-SY5Y CTR1^–/–^	24-well plate	1,0 × 10^4^; 5,0 × 10^4^

### Toxicity Experiments

SH-SY5Y WT and CTR1^–/–^ cell lines were grown and differentiated in 96-well white, clear-bottom plates. Differentiated WT cells were treated with 0–500 μM CuCl_2_ in combination with 0–1 μM DSF (HY-B0240, MedChemExpress), 0–600 μM TU (T8656, Sigma-Aldrich), or 0–25 μM 3-AP (BD01097198, BLDpharm) for 24 h. Differentiated CTR1^–/–^ cells were treated with 0–500 μM CuCl_2_ in combination with 0–800 μM LA (T5625, Sigma-Aldrich) or 0–1 μM ES (SML2651, Sigma-Aldrich) for 24 h. Propidium iodide (PI) (81845, Fluka) staining was used to assess cell viability. A 0.5 mM PI solution in phosphate-buffered saline (PBS; P4417, Sigma) was added to the samples to obtain a final concentration of 2,5 μM. Samples were incubated with PI for 10 min at 37 °C followed by fluorescence measurement using CLARIOstar Plus (BMG LABTECH) with excitation and emission set to 540 and 612 nm, respectively. All experiments were conducted using the same methodology as in our previous study.^7^

### Inductively Coupled Plasma Mass Spectrometry (ICP-MS) Analysis

SH-SY5Y WT and CTR1^–/–^ cells were grown and differentiated in clear-bottom 6-well plates (Greiner Bio One). For ^63^Cu measurements, the cells were treated for 24 h with 5 μM CuCl_2_ in the presence of various concentrations of DSF, TU, 3-AP, ES, and LA. For data normalization, cells were counted after 24-h incubation using a Neubauer cell counter and trypan blue (C10228, Invitrogen). Because the investigated compounds function as copper-binding ligands and ionophores, ICP-MS experiments were performed under copper-supplemented conditions to evaluate their ability to modulate extracellular copper uptake, as their effects depend on the availability of extracellular copper. In the absence of copper supplementation, only redistribution of the limited endogenous cellular copper pool would be expected.

Control and compound-treated cells were collected from 6-well plates into acid-washed 2 mL Eppendorf tubes. Cells were separated from the medium and washed twice with PBS. The supernatant was aspirated, and the cell pellets were stored at −80 °C until the ICP-MS analysis.^7^

Samples were dissolved in 100 μL 68% HNO_3_ and incubated at room temperature for 24 h. After acid digestion, the samples were diluted to 3,4% HNO_3_. Ultrapure Milli-Q-quality water with a resistivity of 18,2 MΩ/cm, produced by a Merck Millipore Direct-Q & Direct–Q UV water purification system (Merck KGaA, Darmstadt, Germany), was used for all sample preparations.

Quantification of ^63^Cu was performed on an Agilent 7800 series ICP-MS instrument (Agilent Technologies, Santa Clara, USA) equipped with an Agilent SPS-4 autosampler for sample introduction. Instrument control and data acquisition were carried out using ICP-MS MassHunter 4.4 software Version C.01.04 from Agilent. ICP-MS analysis was performed in peak-hopping mode, 6 points per peak, 100 scans per replicate, 3 replicates per sample. The instrument was operated in general matrix working mode under the following conditions: RF power 1550 W, nebulizer gas flow 1.05 l/min, plasma gas flow 15 l/min, nebulizer type: MicroMist. Measurements were performed in He mode. The ICP-MS instrument was calibrated using multielement calibration standard solutions in the range of 0.50–50 ppb in 3.4% trace metal grade HNO_3_ (8500–6940, Agilent Technologies, United States) containing Ag, Al, As, Ba, Be, Ca, Cd, Co, Cr, Cs, Cu, Fe, Ga, K, Li, Mg, Mn, Na, Ni, Pb, Rb, Se, Sr, Tl, U, V, Zn. The internal standard for ^63^Cu was ^72^Ge (5188–6525, ICP-MS internal standard mix 1 μg/mL in 2% HNO_3_, Agilent Technologies). All experiments were made using identical methodology as in our previous study.^7^

### Differentiation and Proliferation of SH-SY5Y CTR1^–/–^ Cell Line

SH-SY5Y CTR1^–/–^ cell line was grown and differentiated in clear-bottom 24-well plates. Cells were differentiated according to the protocol described above, with medium supplemented with 20 μM LA, 5 μM CuCl_2_, or a combination of 20 μM LA and 5 μM CuCl_2_ Cells were counted using a Neubauer cell counter with trypan blue (C10228, Invitrogen) and imaged using a Zeiss Axiovert 200 M fluorescence microscope.

### Statistical Analyses

Data visualization and statistical analysis were performed using GraphPad Prism 10 software. Data was analyzed using a one-way analysis of variance (ANOVA) with the post hoc Dunnett’s multiple comparison test (indicated by a continuous line) and paired or unpaired *t* test (indicated by a dotted line) where appropriate. Toxicity and ICP-MS experiments were performed in triplicate and were independently repeated at least three times. SH-SY5Y CTR1^–/–^ cells differentiation and proliferation determination experiment consisted of six independent repeats. Data on graphs are presented as mean ± standard error of the mean (SEM). Statistical significance was indicated as follows: * - *p* ≤ 0.05, ** - *p* ≤ 0.01, *** - *p* ≤ 0.001, and **** - *p* ≤ 0.0001.

## Results

### Copper-Dependent Effects of DSF, TU, and 3-AP on SH-SY5Y WT Cell Viability

Results with DSF and TU were generally consistent with our group’s previous findings and publication, where the toxicity of LA and ES in the presence of copper on SH-SY5Y WT cells was studied.^7^ Additionally, copper alone was not toxic to SH-SY5Y WT cell cultures at concentrations up to 1 mM of CuCl_2_.^7^ TU exhibited only a slight toxic effect, similar to LA, in the presence of 5 μM CuCl_2_. DSF was only slightly toxic at submicromolar concentrations in the presence of 5 μM CuCl_2_, however, at micromolar concentrations, DSF resembled ES in its toxicity (Figure 2).

**Figure 2:**
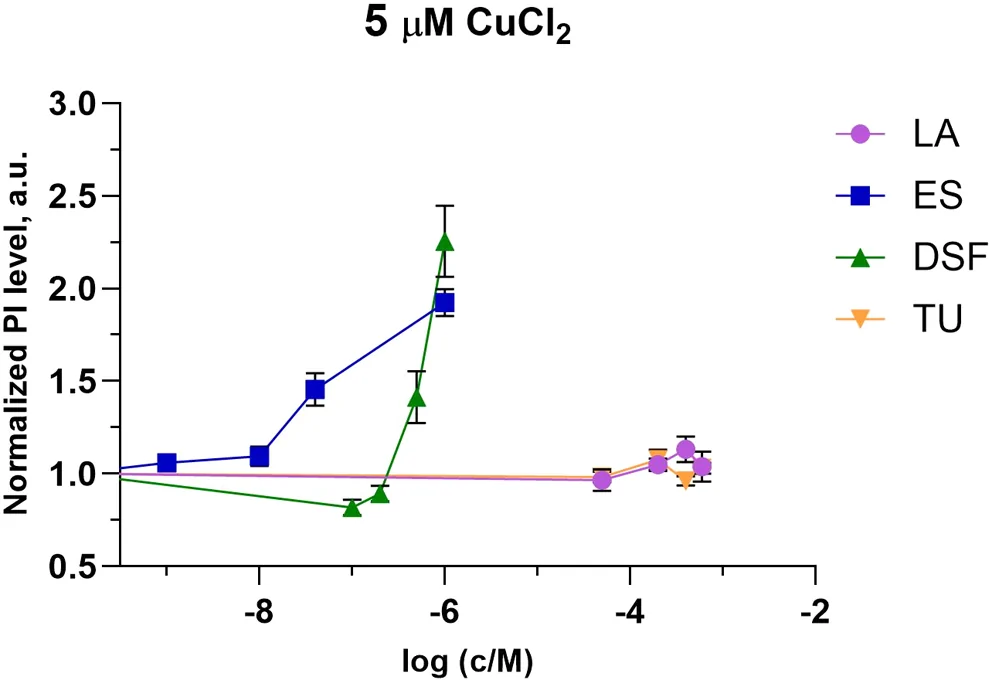
Toxicity of the selected copper-binding ligands to differentiated SH-SY5Y WT cells in the presence of 5 μM CuCl_2_. Cells were incubated for 24 h with different concentrations (20 nM–1 μM) of DSF and TU in the presence of 5 μM CuCl2. c/M is the logarithm of the numeric value of molar concentration. Data with ES and LA is previously published in the article Kirss et al., 2024. PI fluorescence was measured at 612 nm (excitation 540 nm). Data is shown mean ± SEM; *n* = 9–21.

When administered alone, neither DSF nor TU reduced WT cell viability (Supporting Figure 1). TU-associated toxicity was observed only at high combined concentrations (500 μM TU + 600 μM CuCl_2_) (Supporting Figure 2). Nevertheless, minor morphological changes were detectable at 200 μM TU in combination with 500 μM CuCl_2_ where cells looked flatter and rounder (Supporting Figure 3C). In contrast, DSF demonstrated a stronger copper-dependent effect. Treatment with 750 nM DSF in the presence of 5 μM CuCl_2_ resulted in marked cell death (Supporting Figure 4). Additionally, 500 nM DSF combined with 1 μM CuCl_2_ affected cell morphology negatively (Supporting Figure 5). Direct comparison between compounds should be interpreted with caution, as ligand and copper concentrations were optimized individually based on compound-specific toxicity profiles and experimental suitability. Therefore, comparisons presented here are intended to identify general toxicity trends and copper-dependent effects rather than establish quantitative differences in potency between ligands.

Treatment with 3-AP alone reduced cell viability in a concentration-dependent manner (Figure 3A). However, cotreatment with copper eliminated this effect. Exposure to 25 μM 3-AP for 24 h significantly decreased cell viability compared to untreated controls, whereas coadministration with copper restored viability to control levels. Copper concentrations ≥ 25 μM CuCl_2_ fully abrogated 3-AP-induced cytotoxicity (Figure 3B). Due to different toxicity profiles of the compounds used, optimal ligand concentrations were selected individually in the presence of 5 μM CuCl_2_ for ICP-MS studies to mimic physiologically relevant conditions while avoiding overt toxicity. Consequently, the experimental design was intended to assess copper-dependent cellular responses under nontoxic conditions rather than provide a strict head-to-head comparison of compound potency.

**Figure 3:**
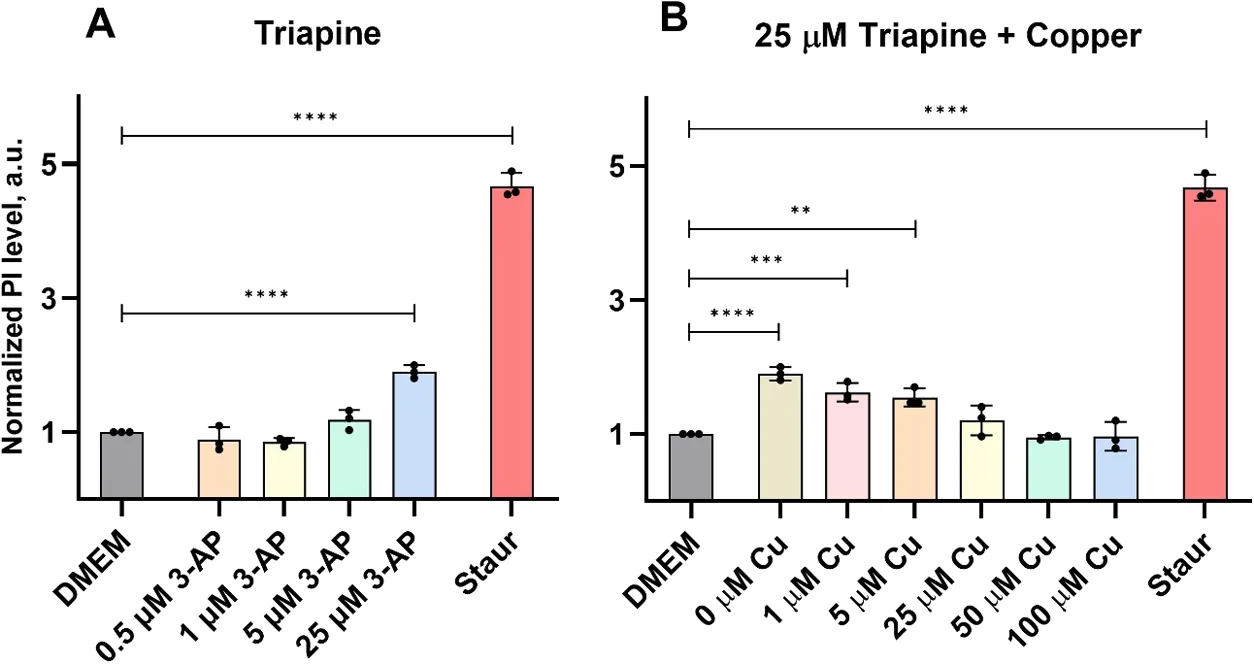
Toxicity of Triapine (3-AP) individually and in the presence of copper to differentiated SH-SY5Y WT cells. (A) Cells were incubated for 24h with 0.5–25 μM 3-AP. (B) Cells were incubated for 24h with 25 μM 3-AP in the presence of 1–100 μM CuCl_2_. PI fluorescence was measured in 612 nm (excitation 540 nm). DMEM was used as a control without any compounds. Staurosporine (staur) was used as a positive control of apoptosis.

### SH-SY5Y CTR1^–/–^ Cells Exhibit Reduced Sensitivity to Copper Cotreatment

The toxicity of LA and ES in the presence of copper has previously been described in SH-SY5Y WT cells.^7^ In the present study, SH-SY5Y CTR1^–/–^ cells were additionally evaluated as a comparative model using identical methodology as in our previous study. To assess copper-associated toxicity, differentiated CTR1^–/–^ cells were incubated for 24 h with increasing concentrations of LA or ES in the presence of CuCl_2_.

For comparative analysis between WT and CTR1^–/–^ cells, 500 μM CuCl_2_ was selected for cotreatment with 0–800 μM LA, and 100 μM CuCl_2_ was selected for cotreatment with 0–1 μM ES. In WT cells, cotreatment with 500 μM CuCl_2_ and 200 μM LA significantly decreased cell viability. In contrast, CTR1^–/–^ cells were not significantly affected under the same conditions (Figure 4A). Similarly, 1 μM ES in combination with 100 μM CuCl_2_ significantly reduced viability in WT cells, whereas no significant effect was observed in CTR1^–/–^ cells (Figure 4B).

**Figure 4:**
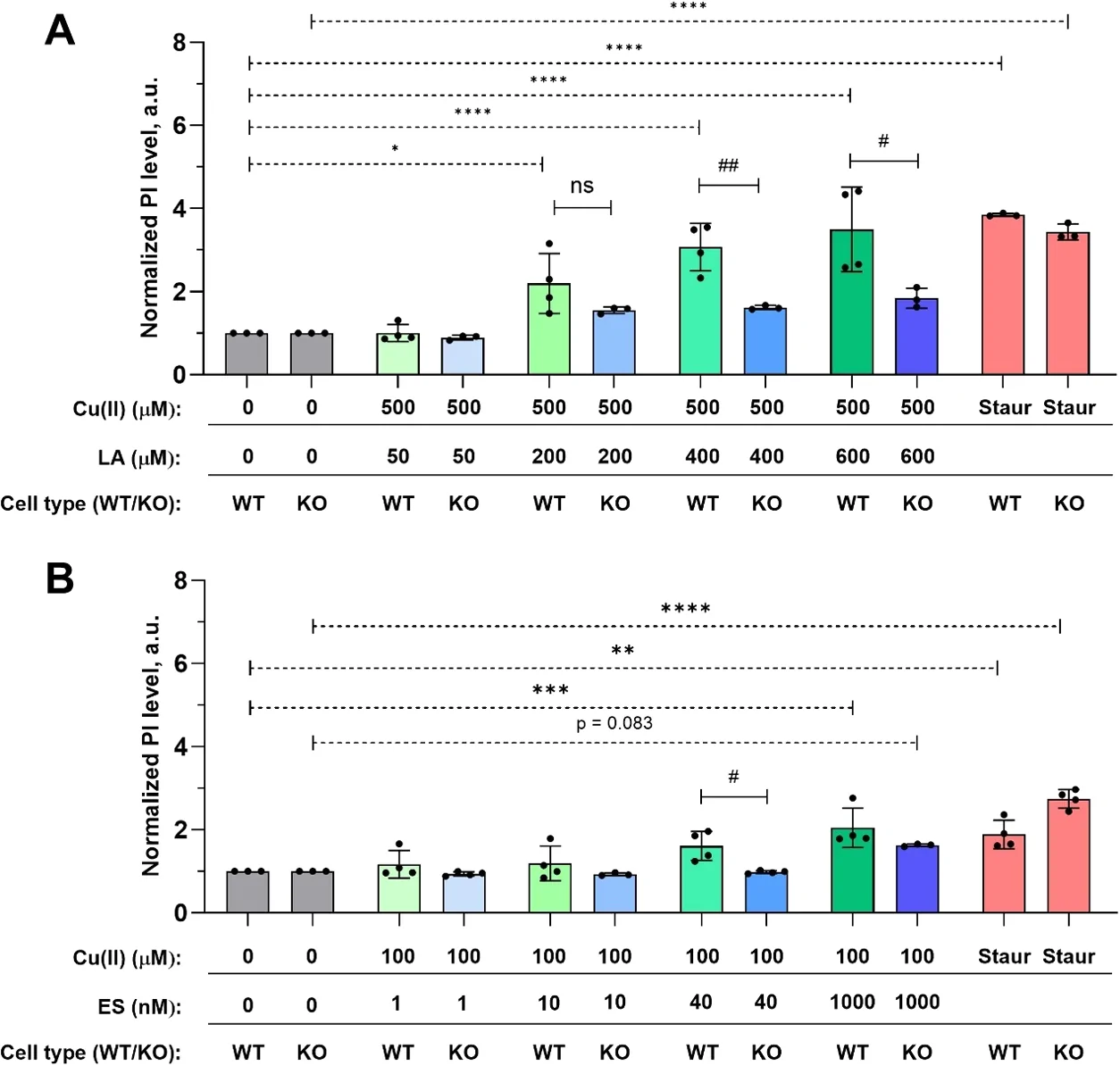
Comparison of copper-induced toxicity with LA or ES on differentiated SH-SY5Y WT and CTR1^–^/^–^(KO) cells. (A) Cells were incubated for 24h with 500 μM CuCl_2_ in the presence of different concentrations of LA (0 – 600 μM LA). (B) Cells were incubated for 24h with 100 μM CuCl_2_ in the presence of different concentrations of ES (0–1 μM ES). PI fluorescence was measured at 612 nm (excitation 540 nm). Staurosporine (Staur) was used as a positive control of apoptosis.

Overall, cosupplementation of LA or ES with CuCl_2_ resulted in a greater reduction of viability in SH-SY5Y WT cells compared to CTR1^–/–^ cells, indicating reduced sensitivity of the CTR1^–/–^ cell line to extracellular copper under the tested conditions.

### α-Lipoic Acid, Elesclomol, and Disulfiram Increase Intracellular Copper Levels in CTR1^–/–^ Cells

LA and ES have been reported to promote copper influx in SH-SY5Y WT cells.^7^ Here we showed that TU and 3-AP did not increase cellular copper content compared to treatment with copper alone. However, DSF was able to increase copper content in WT cells (Figure 5).

**Figure 5:**
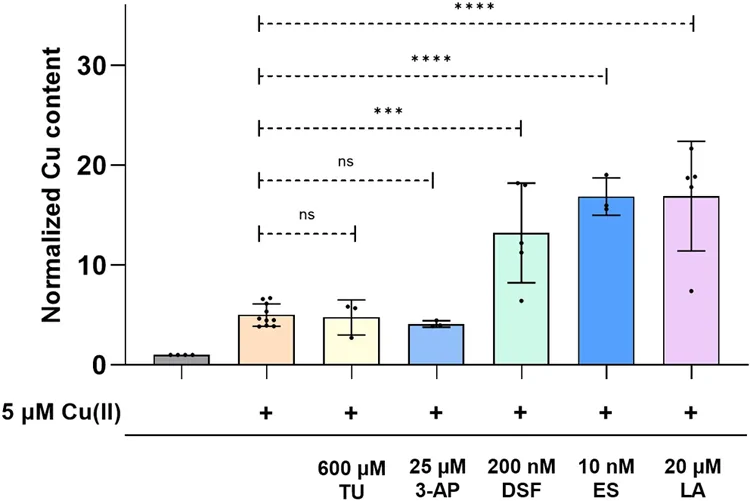
Copper uptake into differentiated SH-SY5Y WT cells following treatment with selected copper-binding ligands. Cells were treated with 600 μM TU; 25 μM 3-AP; 200 nM DSF; 10 nM ES and 20 μM LA in the presence of 5 CuCl_2_ for 24 h. Data are shown as mean ± SEM; *n* = 3–8. Unpaired *t* test with a two-tailed p-value was used for statistical analysis.

To further assess copper uptake independently of CTR1, the same compounds were tested in SH-SY5Y CTR1^–/–^ cells using identical methodology, and intracellular copper levels were compared with previously published WT data (Figure 6A,B).^7^

**Figure 6:**
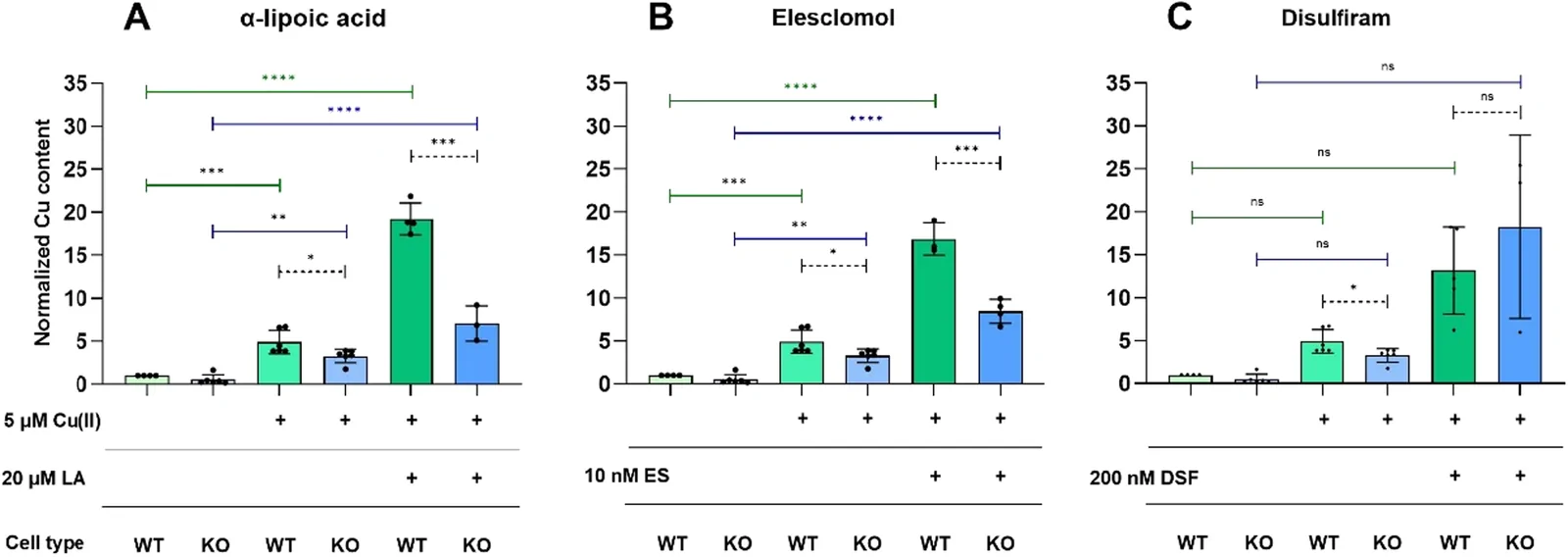
Comparison of the influx of copper into differentiated SH-SY5Y WT and CTR1^–^/^–^(KO) cells following treatment with selected copper-binding ligands. Cells were treated with 20 μM LA (A); 10 nM ES (B) and 200 nM DSF (C) in the presence of 5 CuCl_2_ for 24 h. Data are shown as mean ± SEM; *n* = 3–8. Unpaired *t* test with two-tailed p-value (dotted lines) and ANOVA with the post hoc Dunnett’s multiple comparison test (continuous line) were used for statistical analysis.

Treatment of CTR1^–/–^ cells with 5 μM CuCl_2_ alone increased intracellular copper levels compared to untreated controls (Figure 6). Co-treatment with 20 μM LA and 5 μM CuCl_2_ significantly increased copper content compared to both untreated and copper-only treated CTR1^–/–^ cells (Figure 6A). Similarly, cosupplementation with 10 nM ES significantly elevated intracellular copper levels relative to control and copper-only conditions (Figure 6B). Treatment with 200 nM DSF also significantly increased copper content compared to control and copper-only treated cells (Figure 6C).

To directly compare copper accumulation between cell lines, CTR1^–/–^ data were normalized to WT control values. Treatment with 5 μM CuCl_2_ resulted in significantly lower intracellular copper levels in CTR1^–/–^ cells compared to WT cells (Figure 6). Co-treatment with 20 μM LA or 10 nM ES led to significantly lower copper accumulation in CTR1^–/–^ cells relative to WT cells under the same conditions (Figure 6A, B). In contrast, DSF-treated CTR1^–/–^ cells did not exhibit reduced copper levels compared to WT cells; instead, intracellular copper levels in CTR1^–/–^ cells were comparable to or higher than those observed in WT cells (Figure 6C). Overall, LA, ES, and DSF increased intracellular copper levels in CTR1^–/–^ cells, whereas 3-AP did not enhance copper accumulation beyond copper-only treatment.

### α-Lipoic Acid Cosupplemented with Copper Enhances Proliferation and Differentiation of SH-SY5Y CTR1^–/–^ Cells

By comparing with WT cells, we observed that differentiated SH-SY5Y CTR1^–/–^ cells exhibit lower cell density and shorter neurite-like structures. SH-SY5Y CTR1^–/–^ cells exhibit lower intracellular copper levels compared to WT cells, which may influence cellular growth characteristics. To evaluate whether increasing copper availability affects CTR1^–/–^ cell proliferation and differentiation, cells were differentiated in the presence of 20 μM LA and 5 μM CuCl_2_.

Compared to standard differentiation conditions, cosupplementation with 20 μM LA and 5 μM CuCl_2_ significantly increased the number of CTR1^–/–^ cells (Figure 7A). Treatment with 5 μM CuCl_2_ alone also increased cell number relative to untreated differentiated controls; however, the combination of LA and copper resulted in a further increase in proliferation (Figure 7A).

**Figure 7:**
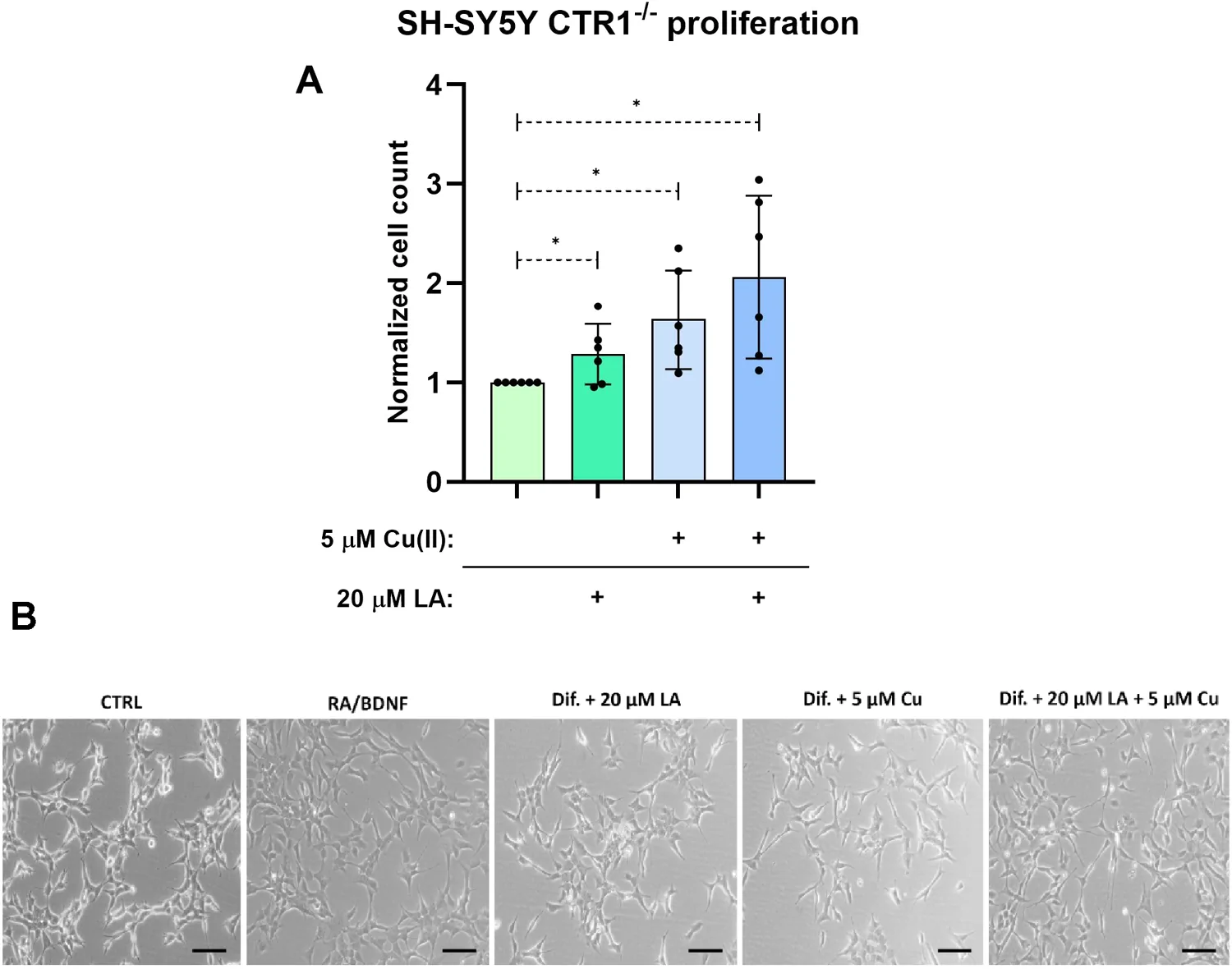
SH-SY5Y CTR1^–/–^ cell culture proliferation and differentiation in the presence of LA and copper. (A) Comparison of cell count after differentiation protocol in the presence of copper and/or LA; (B) bright field images of the cells, scalebar – 100 μm. Data are shown as mean ± SEM; *n* = 6. Unpaired *t* test with two-tailed p-value was used for statistical analysis.

In addition to increased cell numbers, LA and copper cotreatment induced noticeable morphological changes. CTR1^–/–^ cells displayed a more differentiated phenotype, characterized by extended neurite-like structures, resembling the morphology typically observed in differentiated SH-SY5Y WT cells (Figure 7B).

These results demonstrate that cosupplementation with LA and copper enhances both proliferation and differentiation-associated morphological features in SH-SY5Y CTR1^–/–^ cells.

## Discussion

In the present study, we evaluated the cytotoxicity and effects on intracellular copper levels of the copper-binding compounds DSF, TU, and 3-AP, and compared these with LA and ES, which have been investigated previously.^7^ We further investigated the effects of LA, ES, DSF and 3-AP on differentiated SHSY5Y CTR1^–/–^ cells and compared the results with those obtained with SH-SY5Y WT cells.

In the absence of added copper DSF, TU, ES, and LA were nontoxic to the differentiated SH-SY5Y WT cells (Supporting Figure 1).^7^ In the presence of copper, TU showed only a very weak cytotoxic effect (Figure 1) on the WT cells, similar to LA, where cell death was observed only at very high concentrations (Supporting Figures 2 and 3). DSF, an FDA-approved drug for the treatment of alcoholism, was well-tolerated at nanomolar concentrations^18^ and became cytotoxic only at micromolar concentrations. However, ES, a cuproptosis inducer, exhibited substantial cytotoxicity already at nanomolar concentrations (Figure 2). In contrast to these compounds, 3-AP, a well-known chelator for transition metals,^20^ was toxic when applied alone; however, its cytotoxicity was abolished by cotreatment with equimolar copper (Figure 2). It has been demonstrated earlier that the addition of copper in a 1:1 stoichiometry can erase the cytotoxic effect of 3-AP.^20^ Consistently, 25 μM 3-AP-induced toxicity was rescued in the presence of ≥ 25 μM CuCl_2_, whereas lower copper concentrations failed to prevent cell death. This effect is consistent with the known inhibitory action of 3-AP on ribonucleotide reductase leading to apoptosis.^20^

Previously, we demonstrated that coadministration of LA and ES with copper exerts cytotoxic effects on differentiated SH-SY5Y WT cells by increasing intracellular copper levels (Kirss et al., 2024).^7^ In the present study, we investigated their cytotoxic effects and their ability to modulate copper content also in differentiated SH-SY5Y CTR1^–/–^ cells. CTR1^–/–^ cells are reported to exhibit a 70–90% reduction in copper uptake; however, in the absence of CTR1, alternative compensatory copper transport mechanisms may be activated to facilitate copper influx.^27,28^ As expected, cosupplementation with LA or ES and copper resulted in lower cytotoxicity in CTR1^–/–^ cells. The viability of SH-SY5Y WT cells was significantly decreased when cells were incubated with 500 μM CuCl_2_ and 200 μM LA, however, CTR1^–/–^ cells were not significantly affected (Figure 4A). Likewisely, SH-SY5Y WT cells were significantly affected by 1 μM ES treatment cosupplemented with 100 μM CuCl_2_, but in the case of CTR1^–/–^ cells, there was no significant effect (Figure 3B). These results indicate that CTR1^–/–^ cells tolerate higher extracellular copper concentrations, likely due to reduced copper influx. Thereby, copper-induced toxicity during cotreatment with LA occurred at significantly higher LA and copper concentrations compared to ES, supporting the notion that LA facilitates copper uptake into cells in a safer manner.

ICP-MS analysis confirmed compound-specific differences in copper transport. In WT cells, TU and 3-AP did not enhance copper accumulation. At the same time, DSF significantly increased intracellular copper levels when coadministered with 5 μM CuCl_2_, in a dose-dependent manner (Figure 5), reaching levels comparable to ES (Kirss et al., 2024). As DSF exhibits a more favorable toxicity profile than ES, it holds comparable potential to LA for safely normalizing copper metabolism in the context of AD.

In CTR1^–/–^ cells, copper levels increased modestly following CuCl_2_ treatment alone (Figure 6), supporting the presence of alternative copper influx pathways. 3-AP did not enhance copper influx. However, cotreatment with LA or ES and copper increased intracellular copper in CTR1^–/–^ cells, although less efficiently than in WT cells, indicating partial dependence on CTR1. In contrast, DSF restored copper levels in CTR1^–/–^ cells to those observed in WT cells, suggesting that DSF-mediated copper uptake does not rely on CTR1 (Figure 6).

As the morphology of differentiated WT and CTR1^–/–^ cells differed, we also studied the effect of copper supplementation on proliferation and differentiation of this cell line. It is known from the literature that copper deficiency may impair cell proliferation and differentiation.^29^ We found that cosupplementation of copper and LA, which boosts copper levels in CTR1^–/–^ cells, can uplift the cellular proliferation (Figure 7). Moreover, such treatment changed cellular morphology, where the cells transitioned to a more differentiated state, presenting more neurites (Figure 7). Treatment with copper alone did not significantly promote proliferation or differentiation of CTR1^–/–^ cells, likely because compensatory copper transport mechanisms are insufficient to restore intracellular copper levels to normal.

In summary, we evaluated the ability of different copper-binding compounds to induce cytotoxicity in the presence of copper cosupplementation and assessed their capacity to increase intracellular copper levels in SH-SY5Y WT and CTR1^–/–^ cells. Based on the obtained results, we propose that LA and DSF may represent safe candidates for normalizing copper levels in the AD brain, potentially preventing AD progression. Additional studies at the organismal level are needed to provide deeper insight into the therapeutic effects of these compounds and their ability to improve copper distribution in the brain.

## Supplementary Material



## Notes

Additional experimental details and characterization results; and detailed information about toxicity experiments and cell culture images (PDF)
