# Supplementary material for: Distinct Effects of Copper Ionophores on Intracellular Copper and Cell Viability in Wild-Type and CTR1-Deficient SH-SY5Y Cells
Source: ACS Omega. 2026 Aug 10;11(33):49807–14. doi: 10.1021/acsomega.6c04498 (PMC13520559; doi:10.1021/acsomega.6c04498)
Supplement: Supplementary file 1 [file ao6c04498_si_001.pdf]

Distinct effects of copper ionophores on intracellular copper and cell viability in wild type and CTR1-deficient SH-SY5Y cells

Anette Reinapu[1], Sigrid Kirss[1,*], Vello Tõugu[1] and Peep Palumaa[1,]

[1]Department of Chemistry and Biotechnology, Tallinn University of Technology, Akadeemia tee 15, Tallinn 12618, Estonia

∗ Correspondence:
Sigrid Kirss, Department of Chemistry and Biotechnology, Tallinn University of Technology, Akadeemia tee 15, 12618 Tallinn, Estonia. Tel.: +372 56678452; E-mail: sigrid.kirss@taltech.ee

Supporting information

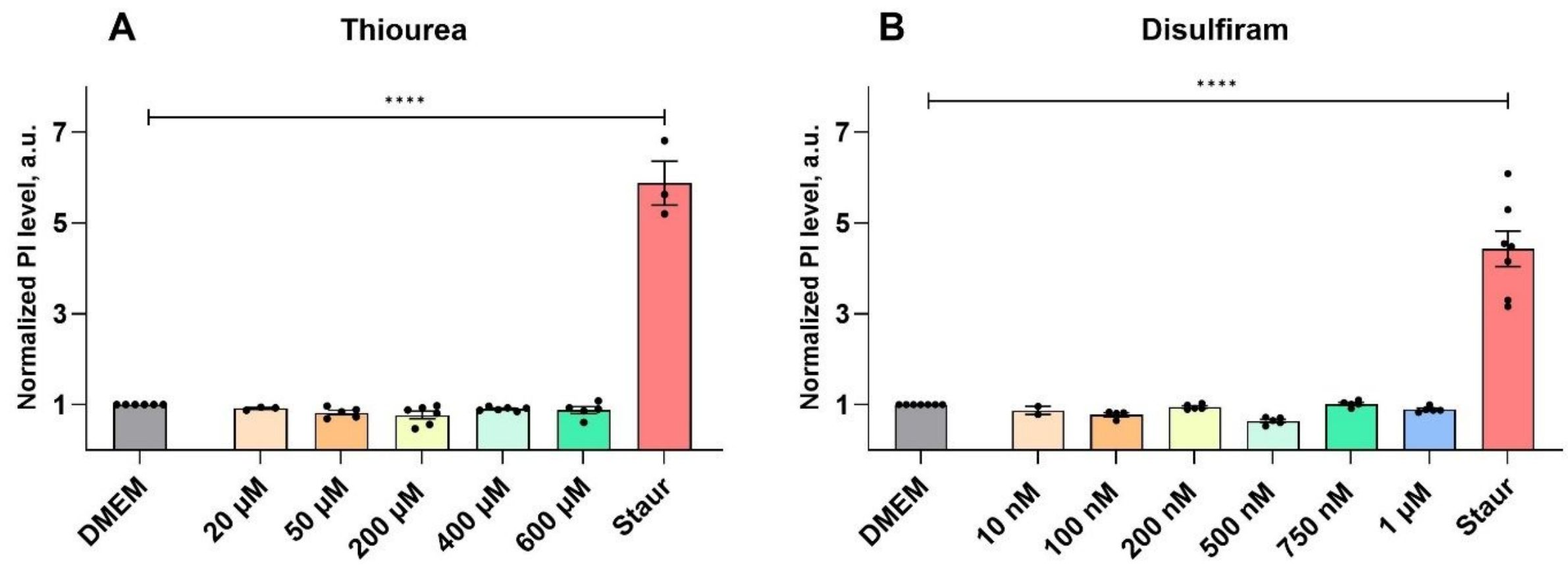

Supplementary figure 1. Toxicity of thiourea (TU) and disulfiram (DSF) individually to differentiated SH-SY5Y WT cells. (A) Cells were incubated for 24h with 20-600 µM or (B) 10 nM – 1 µM DSF. PI fluorescence was measured in 612 nm (excitation 540nm). DMEM was used as a control without any compounds. Staurosporine (staur) was used as a positive control of apoptosis.

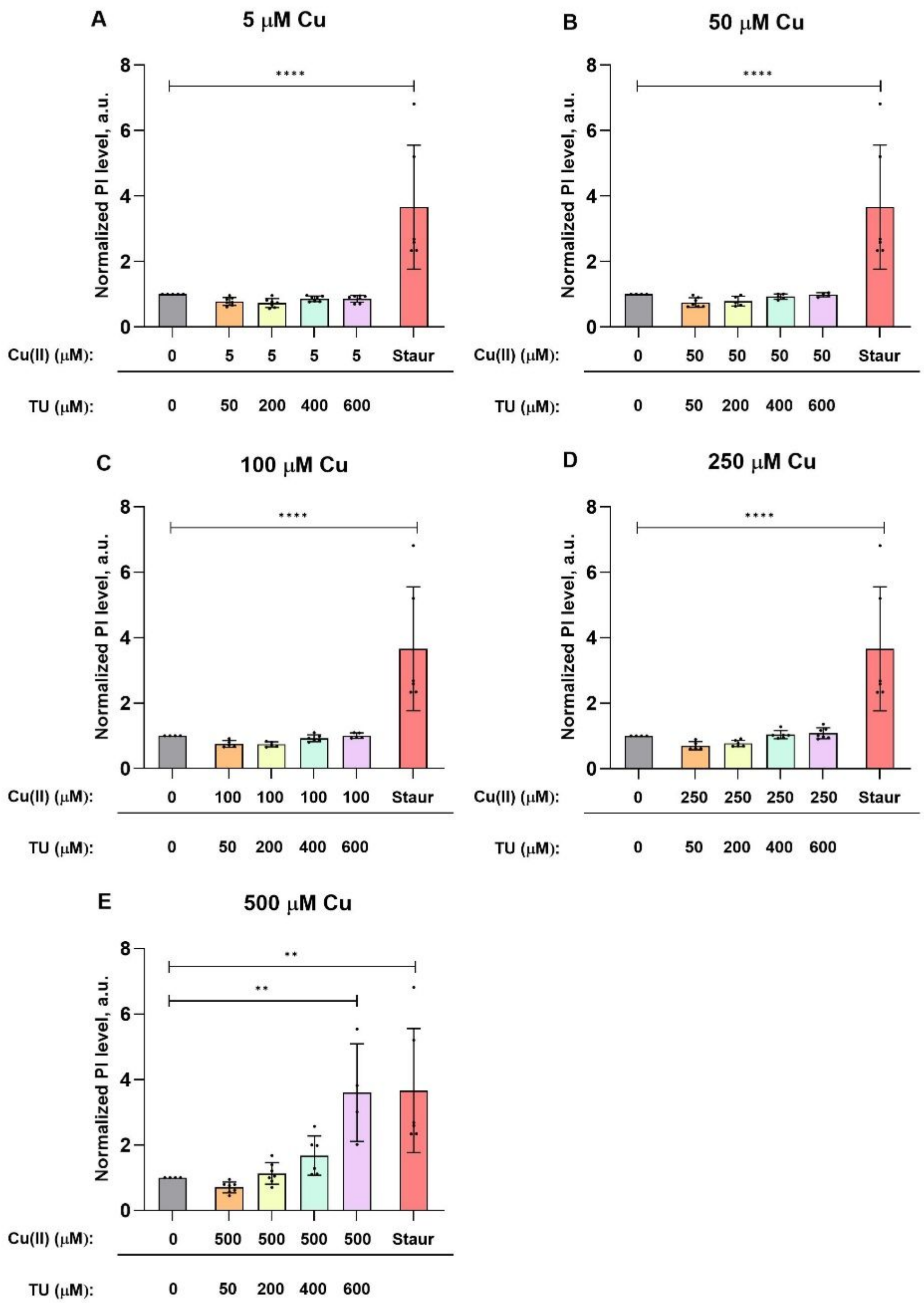

Supplementary Figure 2. Thiourea (TU) and copper co-treatment to differentiated SH-SY5Y WT cells. Cells were incubated for 24 h with different TU concentrations (50 – 600 μM) in the presence of 5 μM $CuCl_2$ (A); 50 μM $CuCl_2$ (B); 100 μM $CuCl_2$ (C); 250 μM $CuCl_2$ (D) and 500 μM $CuCl_2$ (E). PI fluorescence wase measured at 612 nm (excitation 540 nm). Staurosporine (staur) was used as a positive control of apoptosis. Data are shown as mean ± SEM. One-way ANOVA followed by a Dunnett's multiple comparisons test at the 0.05 level was used for statistical analysis. Main effect of treatment ∗∗∗∗ $p < 0.0001$; ∗ $p < 0.05$; n.s., not significant.

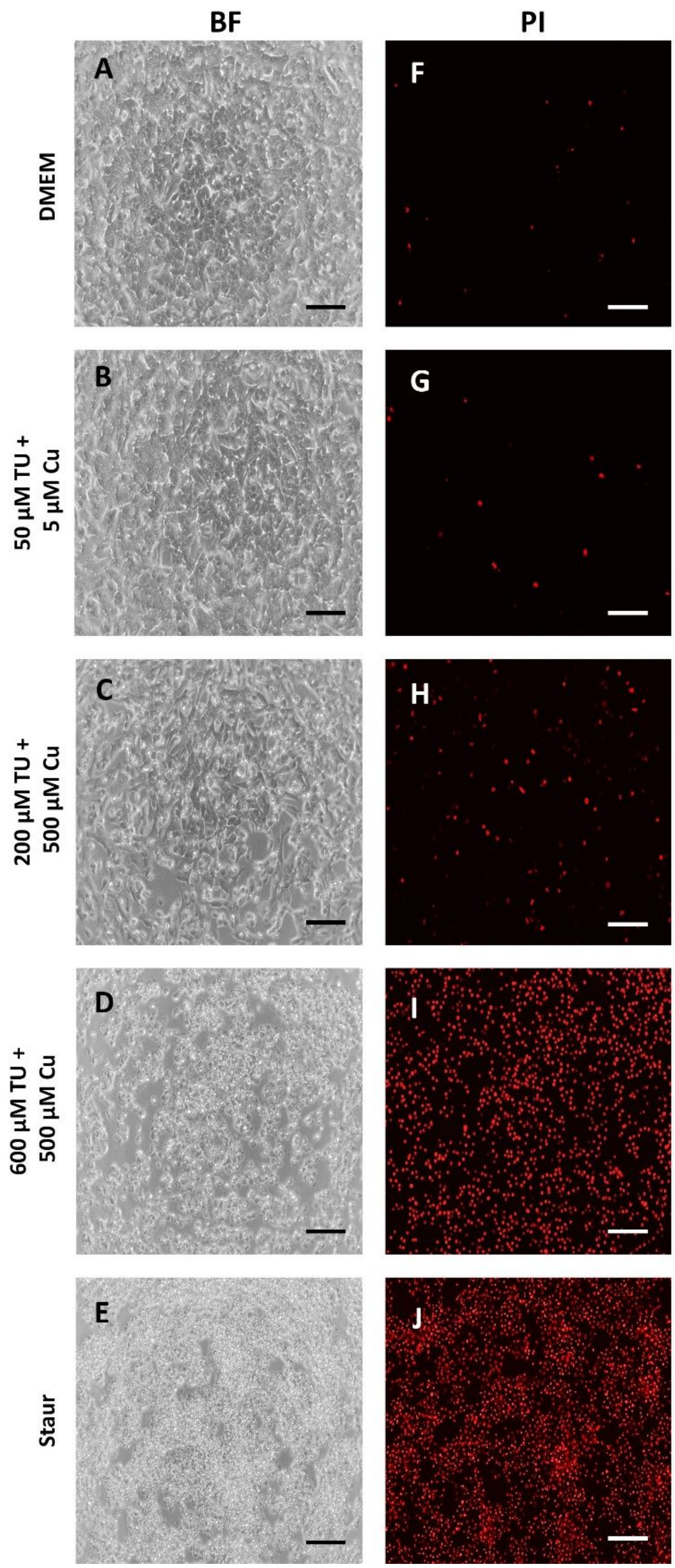
BF
PI
A
B
C
D
E
F
G
H
I
J
DMEM
50 µM TU +
5 µM Cu
200 µM TU +
500 µM Cu
600 µM TU +
500 µM Cu
Staur

Supplementary Figure 3. Bright-field and PI fluorescence images of differentiated SH-SY5Y WT cells after incubation with copper and thiourea (TU) for 24 hours. Bright-field images of the cells (A-E) and PI fluorescence images (F-J). The images of the cells incubated only in media (DMEM) (A, F) and incubation with Staurosporin (Staur) (E, J). Cells incubated with 50 µM TU + 5 µM $CuCl_2$ (B, G); 200 µM TU + 500 µM $CuCl_2$ (C, H); and 600 µM TU + 500 µM $CuCl_2$ (D, I). Scalebar – 100 µm.

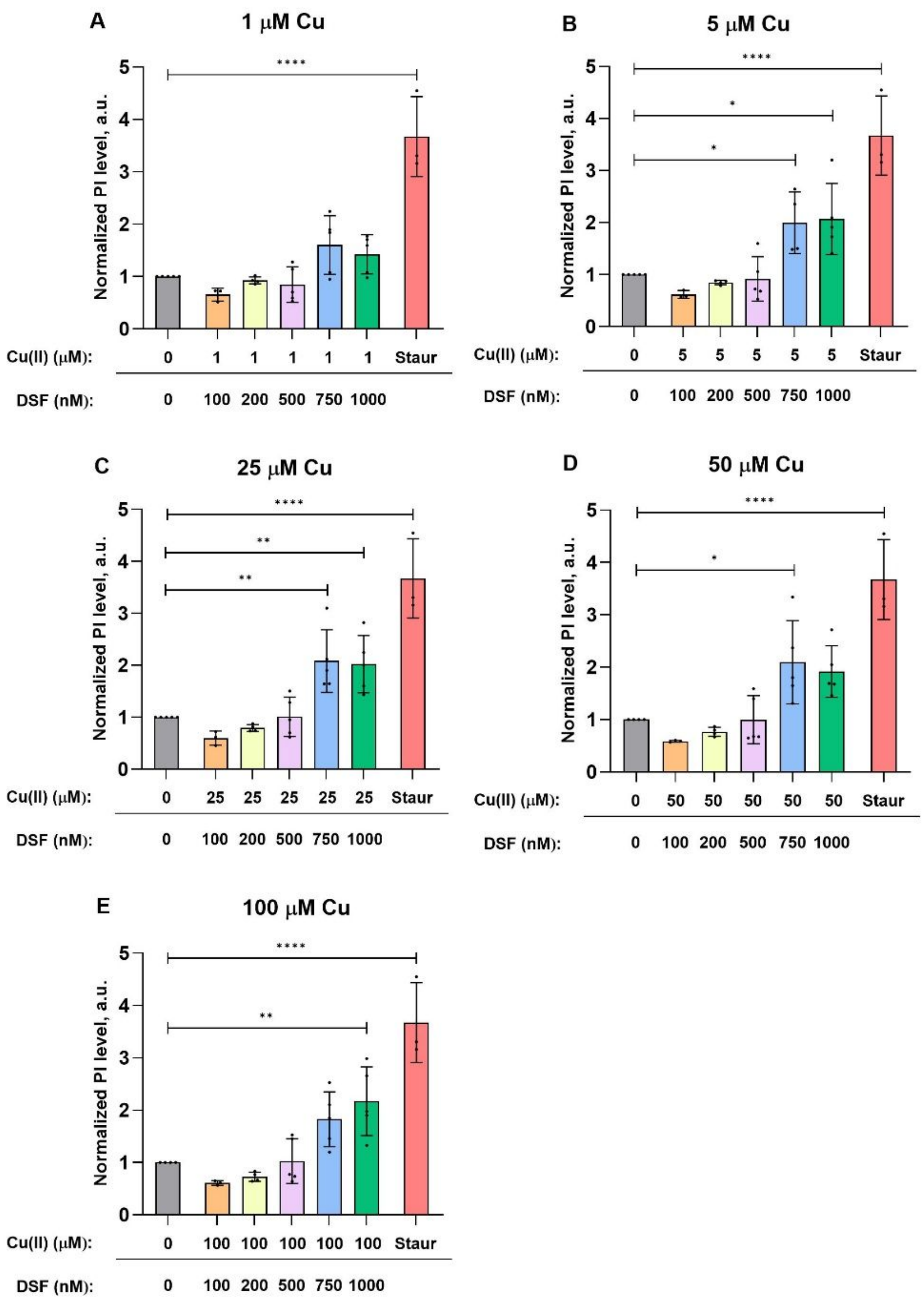

Supplementary Figure 4. Disulfiram (DSF) and copper co-treatment to differentiated SH-SY5Y WT cells. Cells were incubated for 24 h with different DSF concentrations (100 nM – 1 μM) in the presence of 1 μM $CuCl_2$ (A); 5 μM $CuCl_2$ (B); 25 μM $CuCl_2$ (C); 50 μM $CuCl_2$ (D) and 100 μM $CuCl_2$ (E). PI fluorescence wase measured at 612 nm (excitation 540 nm). Staurosporine (staur) was used as a positive control of apoptosis. Data are shown as mean ± SEM. One-way ANOVA followed by a Dunnett's multiple comparisons test at the 0.05 level was used for statistical analysis. Main effect of treatment ∗∗∗∗ $p < 0.0001$; ∗ $p < 0.05$; n.s., not significant.

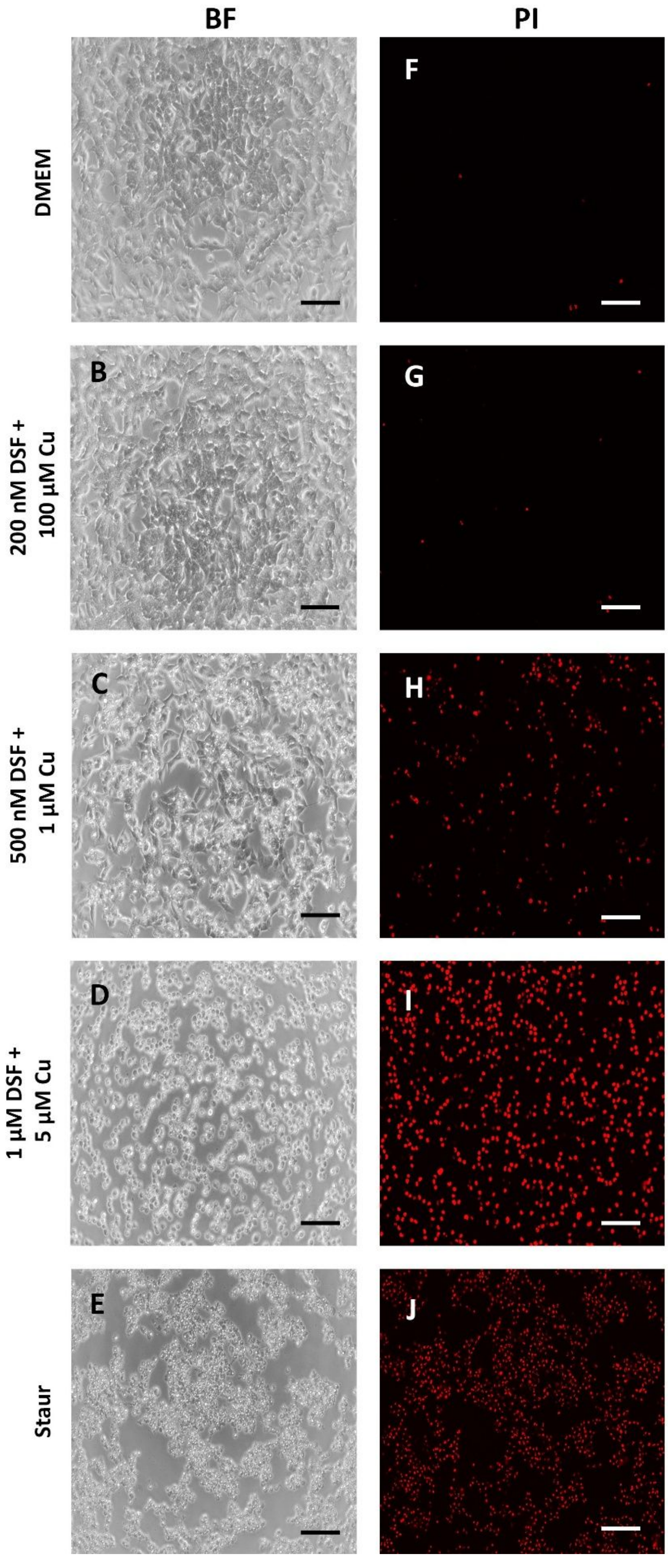

BF
PI
DMEM
200 nM DSF +
100 µM Cu
500 nM DSF +
1 µM Cu
1 µM DSF +
5 µM Cu
Staur
B
C
D
E
F
G
H
I
J

Supplementary Figure 5. Bright-field and PI fluorescence images of differentiated SH-SY5Y WT cells after incubation with copper and disulfiram (DSF) for 24 hours. Bright-field images of the cells (A-E) and PI fluorescence images (F-J). The images of the cells incubated only in media (DMEM) (A, F) and incubation with Staurosporin (Staur) (E, J). Cells incubated with 200 nM DSF + 100 µM $CuCl_2$ (B, G); 500 nM DSF + 1 µM $CuCl_2$ (C, H); and 1 µM DSF + 5 µM $CuCl_2$ (D, I). Scalebar – 100 µm.
